# Assessment of IV-to-PO conversion guideline implementation in pediatric cancer and bone marrow transplant patients after line removal for bloodstream infection

**DOI:** 10.1017/ash.2025.10136

**Published:** 2025-09-11

**Authors:** Adam W. Brothers, Darcy B. Campbell, Victoria J.L. Konold, Michelle M. Palmer, Daniel J. Pak, Matthew P. Kronman, Derry R. McDonald, Jennifer J. Wilkes, Scott J. Weissman

**Affiliations:** 1 Department of Pharmacy, Seattle Children’s Hospital, Seattle, WA, USA; 2 University of Washington School of Medicine, Seattle, WA, USA; 3 Division of Infectious Diseases, Seattle Children’s Hospital, Seattle, WA, USA; 4 Department of Pediatrics, Division of Hematology/Oncology, Ben Towne Center for Childhood Cancer Research, Seattle Children’s Hospital, University of Washington, Seattle, WA, USA

## Abstract

We assessed implementation of a local intravenous-to-enteral antimicrobial transition protocol for pediatric hematology/oncology and bone marrow transplant patients with bacterial or candidal bloodstream infection and central line removal. Among 76 cases, 57 met protocol criteria. Enteral antimicrobials were used in 29 (50.8%) cases meeting eligibility criteria for conversion.

## Background

Historically, antimicrobial treatment for bloodstream infection (BSI) has been delivered strictly by the intravenous (IV) route, although IV therapy can be costly and carries risk of toxicity and other complications.^1^ An emerging body of literature in pediatric and adult patients demonstrates that enteral (PO) antimicrobials reach sufficient concentrations to treat BSI and are safer than IV-only therapy without compromising effectiveness.^2–6^ Despite no mortality association in adult oncology patients with BSI, IV-to-PO conversion is uncommon in immunocompromised pediatric patients.^7,8^ Assessments of safety and effectiveness of PO antimicrobials for BSI in pediatric hematology/oncology (HEMONC) and bone marrow transplant (BMT) patients are lacking.

Conversion to PO antimicrobials was uncommon locally, leading our center to establish guidelines encouraging HEMONC/BMT providers to change from IV-to-PO antimicrobials in their pediatric patients with BSI. This implementation review assesses IV-to-PO adoption among patient cases meeting criteria for enteral antimicrobials. Mortality and infection recurrence are presented as balancing measures.

## Methods

### Study design and setting

In 2019, our freestanding, pediatric, quaternary care hospital implemented a local guideline recommending transition to PO treatment of bacterial or candidal BSI among HEMONC/BMT patients meeting specific criteria, including central line removal (CLR), negative blood cultures, and enteral administration of other medications and nutrition (see Supplemental Material for protocol). This Antimicrobial Stewardship-developed guideline established CLR as a criterion for conversion as it represents substantial source control and provides an opportune time to change antimicrobial routes, although it may not universally be an IV-to-PO requirement. Recommendations were integrated into HEMONC divisional guidelines with re-education occurring annually during divisional Standard Practice training. Education to infectious diseases providers reinforced consideration for IV-to-PO with CLR. Daily audit and feedback by an Antimicrobial Stewardship pharmacist identified eligible cases and encouraged teams to follow guideline recommendations.

This single-center, retrospective assessment describes this guideline implementation, identifying HEMONC/BMT patients with BSI transitioned to PO antimicrobials after CLR. Patients were identified between October 3, 2020, and July 30, 2023. The start date denotes implementation of a new electronic health record for our institution.

BSI-CLR cases were collected for patients admitted to the HEMONC or BMT services when CLR occurred within 14 days of the initial positive blood culture.

Patients were considered eligible for inclusion in this assessment if they were neutropenic or had syndromes that may decrease absorption, although the latter differed from protocol criteria (Supplementary Materials). Infectious diseases consultation was, similarly, not reviewed for inclusion. Patients treated for *Mycobacterium* infections were excluded from analysis because these pathogens frequently require prolonged treatment durations and multiple, concurrent antimicrobials.

Data were collected using Tableau (Seattle, WA) and the Epic health record (Verona, WI). Retrospective chart review was conducted to validate data.

### Outcomes and definitions

The primary outcome was the proportion of eligible BSI-CLR cases among HEMONC/BMT patients with antimicrobial IV-to-PO conversion. Secondary outcomes include infection with any pathogen, recurrence of incident infection, or patient mortality within 30 days of CLR.

PO conversion was defined as ordering an enteral antimicrobial suitable to treat the patient’s BSI within 14 days of CLR. Proportion of PO therapy was calculated as the number of days of enteral antibiotics relative to all antibiotic days of therapy after CLR. Additional variables such as admitting service, unit of care, pathogenic organisms, absolute neutrophil count, and antimicrobial order details were also collected. Absolute neutrophil count was included from the lab drawn closest to the day of CLR, < 200 cells/mm^3^ defining neutropenia. The unit of care was defined as the unit in which the patient experienced their first positive blood culture. Polymicrobial BSI cases identified more than one pathogen on blood culture. IV-to-PO conversion of any antimicrobial used to treat a pathogen within a polymicrobial infection categorized the entire case as a PO conversion.

### Data analysis

Fisher’s exact test was used to compare categorical variables. All analyses were performed using Stata version 14 software (College Station, TX). The institutional review board approved this assessment.

## Results

We examined 76 BSI-CLR cases among 63 HEMONC/BMT patients. Patients with at least 1 BSI case meeting PO eligibility criteria had a median age of 5 years (n = 49 patients, range: 0–20 yrs) compared to 10 years for patients not meeting eligibility (n = 14, range: 0–23 yrs). Other demographic data are available in Table 1.

**Table 1.** Demographics of cases based on protocol eligibility for IV-to-PO conversion

	PO eligible, cases (n=57)	IV only by protocol, cases (n=19)	*p*-value
Primary service			
HEMONC	35 (61.4%)	14 (73.7%)	NS
BMT	22	5	
Neutropenic proximal to CLR			
Neutropenic	14 (24.6%)	9 (47.4%)	*p* = .08
Non-neutropenic	43	10	
Patient care unit			
Non-critical care units	52 (91.2%)	13 (68.4%)	*p* = .02
PICU	5	6	
Pathogens per BSI-CLR case			
Monomicrobial	40 (70.2%)	15 (78.9%)	NS
Polymicrobial	17	4	

PO, enteral; IV, intravenous; HEMONC, hematology-oncology service; BMT, bone marrow transplant service; CLR, central line removal; PICU, pediatric intensive care unit; BSI, bloodstream infection; NS, not significant.

Protocol criteria for IV-to-PO eligibility were met in 57 of 76 cases (75%). Cases not meeting criteria were more likely to occur in the ICU and relative to neutropenia (Table 1).

Conversion to PO antimicrobials occurred during 29 of 57 (50.8%) PO-eligible BSI cases and none of the IV-only episodes (Table 2). The median duration of PO therapy was 6 days (range: 2–22), compared to median total treatment course of 10 days (range: 3–28). Patients changed to enteral antimicrobials received PO agents on 278 of 407 days of therapy after CLR.

**Table 2.** PO conversion and patient outcomes for BSI-CLR cases

	PO eligible, cases (n=57)	IV only by protocol, cases (n=19)	*p*-value
Conversion to PO antimicrobial	29 (50.9%)	0 (0%)	*p* < .0005
Subsequent infection within 30 d	1 (1.8%)	2 (10.5%)	NS
Infection with same pathogen	0	1 (5.3%)	NS
Mortality within 30 d	3 (5.3%)	7 (36.8%)	*p* = .002

PO, enteral; BSI, bloodstream infection; CLR, central line removal; IV, intravenous; NS, not significant.

Re-infection within 30 days of CLR occurred in 3 cases, all among patients who remained on IV antimicrobials. One of those reinfections was recurrence of the incident non-aeruginosa *Pseudomonas* identified prior to CLR.

Ten cases resulted in patient expiration within 30 days of the CLR; mortality was more likely to occur among patients not meeting PO eligibility. Antimicrobials were converted to enteral therapy for one of these cases, treating a *Rothia* and *Granulicatella* BSI, although this patient’s death appeared to be related to uncontrolled *Aspergillus* infection.

Pathogens identified among all BSI cases, as well as enteral agents used for completion of therapy, are available in Supplementary Materials Table 1.

## Discussion

After implementation of an IV-to-PO guideline for BSI antimicrobials among HEMONC/BMT patients at our institution, 50.8% of eligible cases were converted to enteral therapy after CLR. All-cause mortality and infection within 30 days remained low after conversion.

Hesitancy toward using definitive oral therapy may persist, despite proven safety and efficacy in immunocompromised patients, due to long-standing beliefs that IV antimicrobials are more effective than enteral for treating BSI.^9^ Historical, conservative practices, such as reticence to de-escalate empiric febrile neutropenia coverage after identification of a targeted pathogen, may also manifest as hesitancy among providers to prescribe enteral therapy in the setting of neutropenia in pediatric patients. Similar reluctance to use enteral antibiotics was described in an assessment of IV-to-PO therapy in adult oncology patients with gram-negative BSI.^8^

The findings of our quality improvement initiative illustrate that these historical approaches to care for immunocompromised patients are modifiable. Antimicrobial Stewardship teams can facilitate changes in practice through development of shared guidelines with HEMONC/BMT teams, education regarding safety and risks of IV-to-PO versus IV-only practices, and routine surveillance to identify opportunities to make guideline-concordant changes. With only half of eligible patients undergoing IV-to-PO conversion, our findings also highlight that optimizing these practices likely requires iterative cycles of optimization to identify barriers and solutions to improve implementation.

Our requirement for CLR was conservative relative to many IV-to-PO protocols, which may have limited consideration of patient eligibility. However, we believe that this approach improved the willingness of providers to consider enteral therapy instead of obtaining peripheral access to continue IV antimicrobials. This study was also limited by its single-center and retrospective perspectives.

Overall, our intervention was adopted in half of eligible cases, suggesting that opportunities persist to improve IV-to-PO conversion in pediatric HEMONC/BMT patients after CLR for BSI. Among patients who successfully converted to PO antimicrobials, no adverse safety signals were detected.

## Supporting information

10.1017/ash.2025.10136.sm001 Brothers et al. supplementary material Brothers et al. supplementary material

## Supplementary material

The supplementary material for this article can be found at https://doi.org/10.1017/ash.2025.10136.

## References

[1] Kane-Gill SL , Kirisci L , Verrico MM , Rothschild JM. Analysis of risk factors for adverse drug events in critically ill patients. Crit Care Med 2012;40:823–828. 22036859 10.1097/CCM.0b013e318236f473 PMC3581340

[2] Grau D , Clarivet B , Lotthé A , Bommart S , Parer S. Complications with peripherally inserted central catheters (PICCs) used in hospitalized patients and outpatients: a prospective cohort study. Antimicrob Resist Infect Control 2017;6:18. 28149507 10.1186/s13756-016-0161-0 PMC5273851

[3] Spellberg B , Chambers HF , Musher DM , Walsh TL , Bayer AS. Evaluation of a paradigm shift from intravenous antibiotics to oral step-down therapy for the treatment of infective endocarditis: a narrative review. JAMA Intern Med 2020;180:769–777. 32227127 10.1001/jamainternmed.2020.0555 PMC7483894

[4] Davar K , Clark D , Centor RM , et al. Can the future of ID escape the inertial dogma of its past? The exemplars of shorter is better and oral is the new IV. Open Forum Infect Disease 2022;10:706. 10.1093/ofid/ofac706 PMC9853939 36694838

[5] Avent ML , Lee XJ , Irwin AD , et al. An innovative antimicrobial stewardship programme for children in remote and regional areas in Queensland, Australia: optimising antibiotic use through timely intravenous-to-oral switch. J Glob Antimicrob Resist 2022;28:53–58. 34915202 10.1016/j.jgar.2021.11.014

[6] Gunter SG , Wingler MJB , Cretella DA , Wagner JL , Barber KE , Stover KR. Intravenous versus oral step-down for the treatment of *Staphylococcus aureus* bacteremia in a pediatric population. Pharmacy (Basel) 2022;10:16. 35076616 10.3390/pharmacy10010016 PMC8788527

[7] Itoh N , Hadano Y , Saito S , Myokai M , Nakamura Y , Kurai H. Intravenous to oral switch therapy in cancer patients with catheter-related bloodstream infection due to methicillin-sensitive *Staphylococcus aureus*: a single-center retrospective observational study. PLoS One 2018;13:e0207413. 30496212 10.1371/journal.pone.0207413 PMC6264473

[8] Tossey JC , El Boghdadly Z , Reed EE , et al. Oral fluoroquinolones for definitive treatment of gram-negative bacteremia in cancer patients. Support Care Cancer 2021;29:5057–5064. 33594513 10.1007/s00520-021-06063-6

[9] Wald-Dickler N , Holtom PD , Phillips MC , et al. Oral is the new IV. Challenging decades of blood and bone infection dogma: a systematic review. Am J Med 2022;135:369–379. 34715060 10.1016/j.amjmed.2021.10.007 PMC8901545

