# Supplementary material for: Assessment of IV-to-PO conversion guideline implementation in pediatric cancer and bone marrow transplant patients after line removal for bloodstream infection
Source: Antimicrob Steward Healthc Epidemiol. 2025 Sep 11;5(1):e207. doi: 10.1017/ash.2025.10136 (PMC12451790; doi:10.1017/ash.2025.10136)
Supplement: Brothers et al. supplementary material [file S2732494X25101368sup001.docx]

***Supplementary Materials***

ID/HEMONC/BMT Guideline: Use of Enteral Antibiotics for the Treatment of Bacteremia or CLABSI

There are certain clinical scenarios where it may be feasible to administer antimicrobials enterally to treat bacteremia or central-line associated bloodstream infections (CLABSI), in particular where this may decrease the need to put in a new line for IV antibiotics or facilitate the transition outpatient to complete the antibiotic course. Inpatient providers often prefer to treat infectious processes with IV antimicrobials, but broader discussions about early transitions to oral therapy are happening for many conditions including osteomyelitis, urinary tract infections, and pneumonia. ^1^

National guidelines for the treatment of bacteremia or CLABSI do provide some guidance about the feasibility of changing to enteral therapy to complete an antimicrobial course. In the 2009 IDSA Management of Catheter Infections guideline, enteral treatment of certain CLABSI may be possible “when blood cultures have become negative, and signs of sepsis have resolved”.^2^ Additionally, the 2010 IDSA Febrile Neutropenia guideline notes that for patients with a documented infection “The antibiotic spectrum can be appropriately narrowed to specifically treat the defined infection once fever has resolved. In the absence of significant impairment of gastrointestinal function (e.g., nausea, vomiting, diarrhea, malabsorption, poor oral intake), an oral antibiotic regimen may be undertaken to complete the full course of therapy.”^3^

To standardize the patients who may be considered for conversion to enteral therapy, we propose the following criteria:

- Adequate source control has been achieved (e.g., if a line was present and thought to be a source of bacteremia, the infected line has been removed)
- Daily blood cultures have been obtained until cultures remain negative for 48 hours to document sterilization
- Patient has been afebrile for 24 hours
- Infectious Diseases consult service is not concerned for an endovascular focus of infection
- Patient is hemodynamically stable
- Patient not at risk for decreased enteral absorption (e.g., short gut, mucositis, gut GVHD)
- The organism is susceptible to a first-line antimicrobial with high oral bioavailability (>70% per Micromedex ADME data, see Appendix A)

Exclusion criteria: patient age <3 months; central line remains in place; dialysis catheter as source of infection; positive blood culture after the line has been removed; evidence of meningitis or endocarditis. Note: Other condition-specific guidelines or pathways may supersede these criteria.

References

1. Kronman MP, Hersh AL, Newland JG, et al. Getting over our inpatient oral antibiotic aversion. Pediatrics. 2018; 142: e20181634.
2. Mermel LA, Allon M, Bouza E, et al. Clinical practice guidelines for the diagnosis and management of intravascular catheter-related infection: 2009 update by the Infectious Diseases Society of America. Clin Infect Dis. 2009;49:1-45.
3. Freifeld AG, Bow EJ, Sepkowitz KA, et al. Clinical practice guideline for the use of antimicrobial agents in neutropenic patients with cancer: 2010 update by the Infectious Diseases Society of America. Clin Infect Dis. 2011;52:e56-e93.

Appendix A. Table of High Oral-Bioavailability Antimicrobials (>70% bioavailable, per Micromedex)

| Oral products | Intravenous product, if different from oral product |
| --- | --- |
| amoxicillin | ampicillin |
| amoxicillin-clavulanic acid | ampicillin-sulbactam |
| cephalexin | cefazolin |
| ciprofloxacin |  |
| clindamycin |  |
| doxycycline |  |
| levofloxacin |  |
| linezolid |  |
| metronidazole |  |
| minocycline |  |
| moxifloxacin |  |
| rifampin |  |
| trimethoprim-sulfamethoxazole |  |
| fluconazole |  |

**Supplementary Table 1. Organisms Identified from Bloodstream Infection Episodes with Central Line Removal and Enteral Antimicrobials Used for Treatment Among HEMONC/BMT Patients**

|  |  | **BMT** | | **HEMONC** | | **Total** |
| --- | --- | --- | --- | --- | --- | --- |
| **Organism** | **PO agent** | **IV** | **PO switch** | **IV** | **PO switch** |  |
| ***S. epidermidis*** | | | | | | **16** |
|  | linezolid |  | 1 |  | 2 |  |
|  | cephalexin |  |  |  | 2 |  |
|  | levofloxacin |  | 1 |  |  |  |
|  | IV only | 4 |  | 6 |  |  |
| ***Klebsiella* spp** | | | | | | **14** |
|  | sulfa-trim |  | 3 |  |  |  |
|  | amox-clav |  |  |  | 1 |  |
|  | ciprofloxacin |  | 1 |  |  |  |
|  | IV only | 2 |  | 7 |  |  |
| ***S. aureus*** | | | | | | **14** |
|  | cephalexin |  | 1 |  | 2 |  |
|  | linezolid |  | 1 |  |  |  |
|  | IV only | 2 |  | 8 |  |  |
| ***Candida* spp** | | | | | | **13** |
|  | fluconazole |  | 1 |  | 5 |  |
|  | IV only | 1 |  | 6 |  |  |
| ***Enterobacter* spp** | | | | | | **10** |
|  | ciprofloxacin |  | 3 |  |  |  |
|  | sulfa-trim |  | 1 |  |  |  |
|  | IV only | 3 |  | 3 |  |  |
| ***E. faecalis*** | | | | | | **10** |
|  | amoxicillin |  | 3 |  |  |  |
|  | IV only | 3 |  | 4 |  |  |
| ***Pseudomonas* spp** | | | | | | **8** |
|  | ciprofloxacin |  | 2 |  | 1 |  |
|  | levofloxacin |  | 2 |  |  |  |
|  | IV only | 1 |  | 2 |  |  |
| ***E. coli*** | | | | | | **8** |
|  | sulfa-trim |  | 1 |  |  |  |
|  | IV only | 1 |  | 6 |  |  |
| ***Staph* spp*** | | | | | | **6** |
|  | levofloxacin |  | 1 |  |  |  |
|  | IV only | 1 |  | 4 |  |  |
| ***Stenotrophomonas* sp** | | | | | | **3** |
|  | sulfa-trim |  | 1 |  | 1 |  |
|  | minocycline |  |  |  | 1 |  |
| ***Serratia* sp** | | | | | | **3** |
|  | IV only | 2 |  | 1 |  |  |
| ***Pantoea* sp** | | | | | | **2** |
|  | levofloxacin |  | 1 |  | 1 |  |
| ***Rothia* sp** | | | | | | **2** |
|  | amoxicillin |  |  |  | 1 |  |
|  | linezolid |  | 1 |  |  |  |
| ***Enterococcus* spp**** | | | | | | **2** |
|  | IV only | 1 |  | 1 |  |  |
| ***Bacillus* sp** | | | | | | **1** |
|  | levofloxacin |  | 1 |  |  |  |
| ***Chryseobacterium* sp** | | | | | | **1** |
|  | levofloxacin |  |  |  | 1 |  |
| ***Gordonia* sp** | | | | | | **1** |
|  | ciprofloxacin |  |  |  | 1 |  |
| ***Granulicatella*  sp** | | | | | | **1** |
|  | linezolid |  | 1 |  |  |  |
| ***Achromobacter* sp** | | | | | | **1** |
|  | IV only |  |  | 1 |  |  |
| ***Acinetobacter* sp** | | | | | | **1** |
|  | IV only |  |  | 1 |  |  |
| ***Citrobacter* sp** | | | | | | **1** |
|  | IV only | 1 |  |  |  |  |
| ***Lactobacillus* sp** | | | | | | **1** |
|  | IV only |  |  | 1 |  |  |
| ***Microbacterium* sp** | | | | | | **1** |
|  | IV only |  |  | 1 |  |  |
| **viridans-group *Streptococcus*** | | | | | | **1** |
|  | IV only |  |  | 1 |  |  |
| **Grand Total** |  | **22** | **27** | **53** | **19** | **121** |

BSI, bloodstream infection; HEMONC, Hematology-Oncology service; BMT, Bone Marrow transplant service; IV, intravenous; PO, enteral; spp, several species – not individually identified; sp – single species, not identified; sulfa-trim, sulfamethoxazole-trimethoprim; amox-clav, amoxicillin-clavulanic acid

* non-*S. epidermidis* and non-*S. aureus* species

** non-*E. faecalis* species
